# Polynucleotide phosphorylase: Not merely an RNase but a pivotal post-transcriptional regulator

**DOI:** 10.1371/journal.pgen.1007654

**Published:** 2018-10-11

**Authors:** Todd A. Cameron, Lisa M. Matz, Nicholas R. De Lay

**Affiliations:** 1 Department of Microbiology and Molecular Genetics, McGovern Medical School, University of Texas Health Science Center, Houston, Texas, United States of America; 2 MD Anderson Cancer Center UTHealth Graduate School of Biomedical Sciences, University of Texas Health Science Center, Houston, Texas, United States of America; Ohio State University, UNITED STATES

## Abstract

Almost 60 years ago, Severo Ochoa was awarded the Nobel Prize in Physiology or Medicine for his discovery of the enzymatic synthesis of RNA by polynucleotide phosphorylase (PNPase). Although this discovery provided an important tool for deciphering the genetic code, subsequent work revealed that the predominant function of PNPase in bacteria and eukaryotes is catalyzing the reverse reaction, i.e., the release of ribonucleotides from RNA. PNPase has a crucial role in RNA metabolism in bacteria and eukaryotes mainly through its roles in processing and degrading RNAs, but additional functions in RNA metabolism have recently been reported for this enzyme. Here, we discuss these established and noncanonical functions for PNPase and the possibility that the major impact of PNPase on cell physiology is through its unorthodox roles.

## Author summary

Widely distributed among bacteria and eukaryotes, including humans, polynucleotide phosphorylase (PNPase) is a critical enzyme in RNA metabolism that functions in most organisms as a 3ʹ to 5ʹ exoribonuclease. In bacteria, inactivation of the gene encoding PNPase results in a wide range of consequences, including impaired growth, diminished stress responses, and loss of virulence. In mammals, PNPase has an essential role in mitochondrial function. Mutations in the gene encoding the human PNPase (hPNPase) that reduce its activity can lead to hereditary hearing loss, encephalomyopathy, severe axonal neuropathy, delayed myelination, and Leigh syndrome. In this review, we highlight both the canonical and unorthodox activities that have been reported for PNPase. Specifically, we examine its role in bacterial mRNA and rRNA decay, RNA processing, and small regulatory RNA (sRNA) degradation and stabilization. Furthermore, we explore the recently reported findings on the function of hPNPase in mitochondrial RNA import and degradation and cytoplasmic mRNA and noncoding RNA decay. Despite being discovered more than six decades ago, we are still only beginning to grasp the breadth of mechanisms by which the enzymatic activities of PNPase contribute to cellular and organismal physiology.

## Introduction

In 1955, Grunberg-Manago and Ochoa reported that the enzyme polynucleotide phosphorylase (PNPase) from the gram-negative bacterium *Azobacter vinelandii* not only synthesized polynucleotides from nucleotide diphosphates but also catalyzed the reverse reaction, the phosphorolysis of polynucleotides [1, 2]. Heppel and colleagues then demonstrated that these polynucleotides were RNA [3]. PNPase was subsequently used to generate RNA of specific sequences that were employed to decipher the genetic code [4–9].

PNPase is a highly conserved enzyme that is widely distributed among bacteria and eukaryotes, except single-cell eukaryotes such as yeast and trypanosomes [10]. While PNPase can act as a polynucleotide or poly(A) polymerase in bacteria such as *Escherichia coli* [11] or in plant chloroplasts [12], it predominantly functions as a 3ʹ to 5ʹ exoribonuclease in most organisms. PNPase plays critical roles in both bacteria and mammals. Deletion of the gene encoding PNPase from bacteria typically results in a pleiotropic phenotype including increased sensitivity to stressors [13–19] and reduced virulence [14, 20–24]. Moreover, PNPase appears to have an essential function in mammals [25], and mutations that reduce its activity result in mitochondrial disorders in humans [26–29].

In this review, we examine the post-transcriptional regulatory roles of PNPase in bacteria and humans. In bacteria, these include bulk mRNA decay, rRNA degradation, and its paradoxical function in promoting small regulatory RNA (sRNA) stability and function (Table 1). These sRNAs in turn control gene expression by altering mRNA transcription, stability, or translation, placing PNPase in a pivotal position to regulate vast gene networks. We also highlight our emerging understanding of the functions that PNPase performs in humans, including transporting RNA into mitochondria, processing mitochondrial RNAs, and degrading sRNAs called microRNAs (miRNAs) that regulate mRNA translation and/or stability. Finally, we discuss how the major impacts of PNPase on cell physiology may be due to its unconventional roles in RNA metabolism.

**Table 1:** Roles and partners of bacterial and human PNPase.

	Context	Localization	Role
**Bacteria**	PNPase alone	Cytoplasm	• General mRNA decay • tRNA and rRNA maturation • Poly(A) and heteropolymeric tail synthesis
	+ RNase E or RNase Y degradosome	Cytoplasmic membrane	• General mRNA decay • Highly structured RNA decay
	+ RNA helicase (RhlB)	Cytoplasm	• rRNA decay • Highly structured RNA decay • tRNA maturation
	+ Rsr and Y-RNA	Cytoplasm	• rRNA decay
	+ Hfq and sRNA	Cytoplasm	• sRNA-mediated gene regulation
**Humans**	PNPase + other proteins?	Mitochondrial IMS	• Mitochondrial RNA import
	+ RNA helicase (SUV3)	Mitochondrial matrix	• Mitochondrial RNA decay
	Apoptosis, overexpression	Cytoplasm	• miRNA, mRNA and polyadenylated noncoding RNA decay

**Abbreviations:** Hfq, host factor for phage Q_β_; hPNPase, human PNPase; IMS, inner membrance space; miRNA, microRNA; PNPase, polynucleotide phosphorylase; Rsr, Ro sixty-related protein; sRNA, small regulatory RNA; SUV3, suppressor of Var1, 3.

### Running PNPase in reverse: Phosphorolysis of RNA by PNPase

In bacteria and eukaryotes, PNPase has an important function in RNA decay. PNPase catalyzes the processive degradation of single-stranded RNA in the 3ʹ to 5ʹ direction using inorganic phosphate as the nucleophile to attack the 3ʹ phosphodiester bond releasing a ribonucleoside diphosphate (NDP). A metal cofactor, Mg^2+^ or Mn^2+^, is required to stabilize the transition state of the phosphate during the reaction [30]. To degrade RNA efficiently, PNPase must bind a single-stranded stretch of RNA at least six nucleotides in length at the 3ʹ terminus [31, 32]. PNPase subsequently degrades RNA in a stepwise motion, rapidly removing discrete segments of 6 to 7 nucleotides between short pauses [33]. A stem loop structure in an RNA substrate can act as a roadblock that halts degradation by PNPase. In vitro studies using a set of GC-rich RNA hairpins of varying length followed by single-stranded sequences demonstrated that a hairpin with a stem as short as seven base pairs inhibited degradation by PNPase [32]. However, PNPase also rapidly degrades some natural hairpins such as the Rho-independent terminators that end many sRNAs [34]. Thus, while very stable hairpins can block the exoribonucleolytic activity of PNPase, merely resulting in 3ʹ end trimming [35], this enzyme can degrade many natural double-stranded RNAs provided that a single-stranded region is present at the 3ʹ end to initiate degradation. This balance between degradation and inhibition is important for maturation of some tRNA transcripts, in which PNPase removes Rho-independent terminators but stops short of the CCA determinants [36–39]. Likewise, structural features of the 16S rRNA preribosomal particle are likely important for preventing excessive 3ʹ trimming of the 16S rRNA by PNPase and other exoribonucleases during its maturation [40, 41].

**Fig 1:**
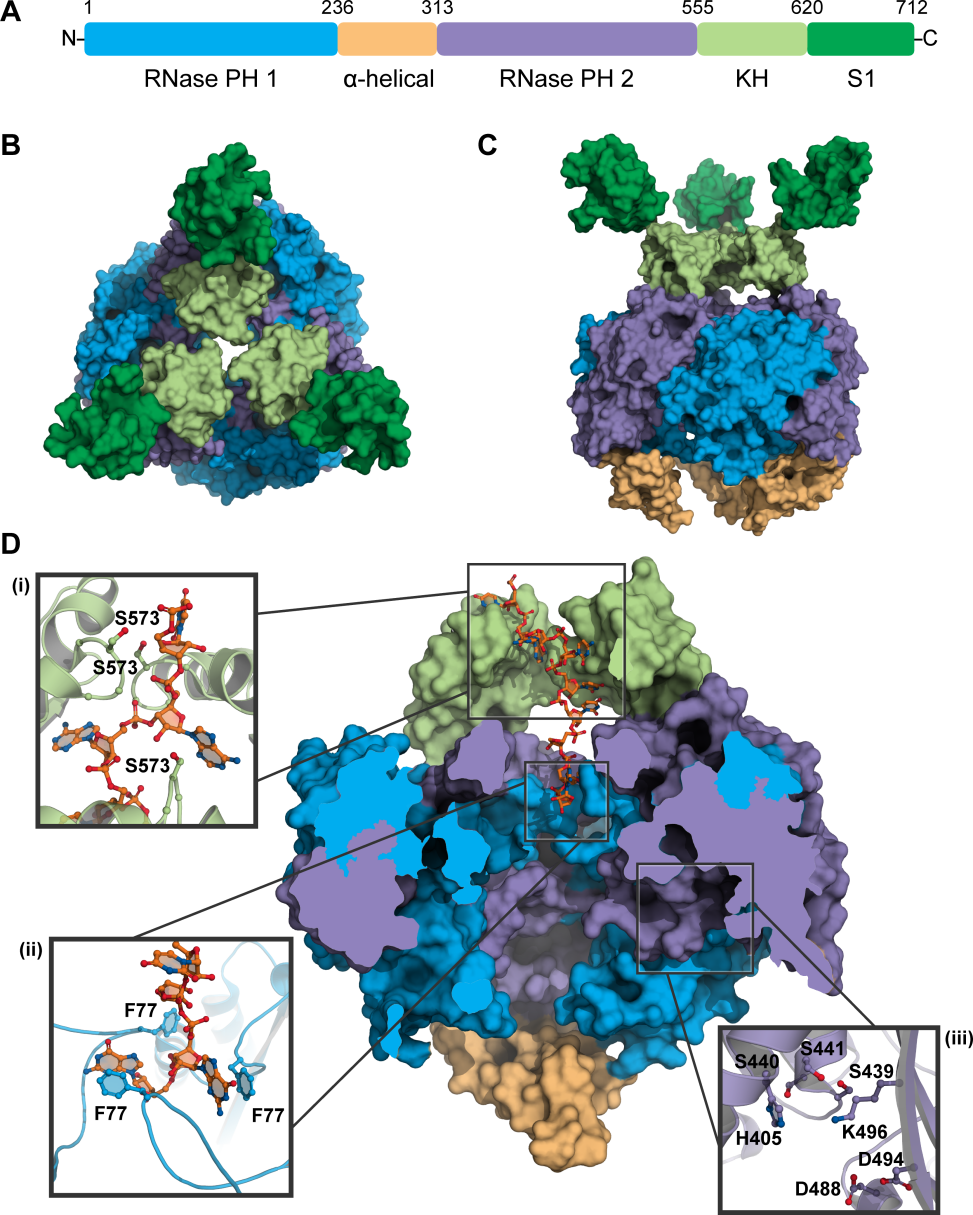
Domains and structure of PNPase. (A) Domain organization of PNPase, using the *C*. *crescentus* PNPase crystal structure as a reference [44]. The complete PNPase trimer viewed from the (B) top and (C) side, with domains colored the same as in (A). (D) A cut-away view of the RNA-bound trimer interior of the PNPase trimer bound to an RNA substrate (orange). Ball and stick structures depict nitrogen atoms as blue and oxygen atoms as red. Inset panels illustrate (i) the interactions between the GSGG loop of each KH domain with the RNA backbone, (ii) base stacking interactions between phenylalanine residues of the FFRR loops and several RNA bases, and (iii) the active site residues that bind phosphate and the metal cofactor. PNPase, polynucleotide phosphorylase.

Crystal structures for PNPase from *Streptomyces antibioticus* [42], *E*. *coli* [30, 43], *Caulobacter crescentus* [44], and *Homo sapiens* [45] have been solved, and much of the substrate specificity and activity of PNPase can be assigned to distinct structural and organizational features of this enzyme. Each PNPase monomer consists of two RNase PH-like domains, which are separated by an α-helical domain and are followed by a KH and S1 domain (Fig 1A). As a functional enzyme, PNPase is assembled into a torus-shaped trimer in which alternating RNase PH-like subunits and α-helical domains form a central ring from which the KH/S1 domains extend outward (Fig 1B and 1C) [30, 42–44]. The first RNase PH-like domain contributes to RNA and NDP binding, and the second domain additionally possesses enzymatic activity [43, 46]. The active site is positioned in a shallow groove along the inner rim of the trimer (Fig 1D, panel iii), and a constriction point formed by the FFRR loop at the entrance of the core ring creates a pore that only allows access by single-stranded RNA (Fig 1D) [43]. Catalytic activity of the central ring is facilitated by the other domains; the α-helical domain appears to regulate access of phosphate or NDP to the active site [30, 47, 48], and the KH and S1 domains each contribute to capturing and binding RNA substrates [44, 49]. Additionally, the KH domain imparts RNA directionality through the interactions of the conserved GSGG loop with the RNA backbone [44]. Finally, the processivity of PNPase is attained through its ring-like structure that retains RNA substrates via multiple RNA-binding interactions, including hydrogen binding between the GSGG loops of the KH domains and the RNA phosphate backbone (Fig 1D, panel i) and base stacking interactions between the aromatic phenylalanines in the conserved FFRR loops and a ribonucleotide base (Fig 1D, panel ii) [44].

### PNPase as a component of the bacterial RNA degradosome

**Fig 2:**
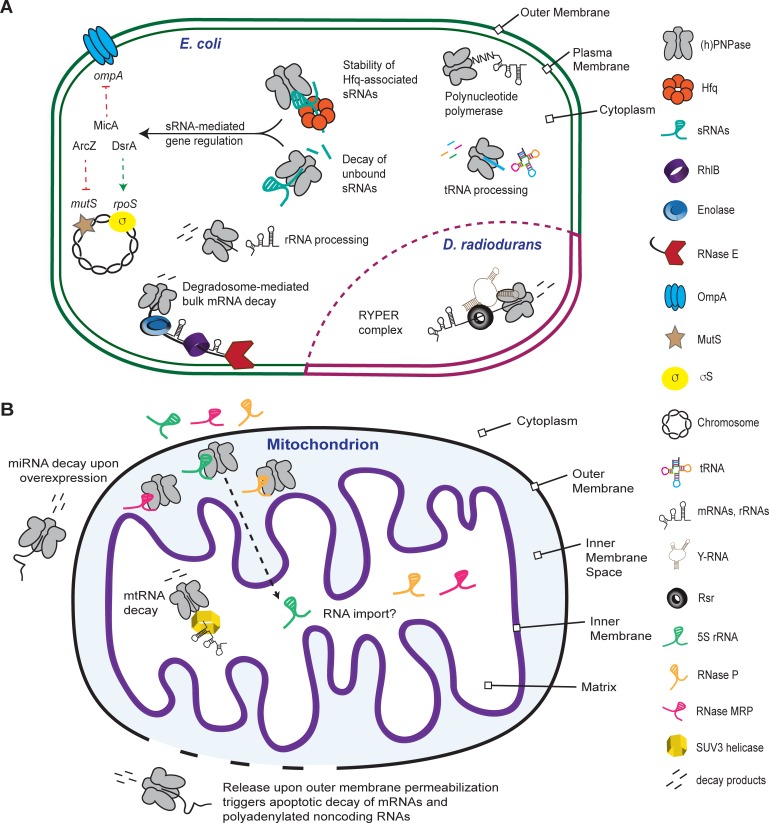
The numerous roles performed by PNPase. Schematic showing the functions performed by PNPase in bacteria and mitochondria. (A) In *E*. *coli* and other gram-negative bacteria, PNPase functions as part of the RNA degradosome in bulk mRNA decay but also operates independently of this machine to process tRNAs and rRNAs, add polynucleotide tails to RNAs, and modulate sRNA stability. PNPase binds Hfq-bound sRNAs but only degrades unbound sRNAs, which impacts both positive and negative sRNA-mediated gene regulation in *E*. *coli*. In *Deinococcus radiodurans*, PNPase forms a complex with Rsr mediated via Y-RNAs that degrades misfolded rRNAs. (B) The locations and functions of hPNPase are controversial, but under normal cellular conditions, hPNPase is mainly localized to the IMS where it is able to facilitate translocation of 5S rRNA, *RNase P* RNA, and possibly *RNase MRP* RNA into the mitochondrial matrix. Within the mitochondrial matrix, hPNPase degrades mitochondrial RNA and plays a role in mitochondrial DNA maintenance. Release into the cytoplasm upon overexpression or permeabilization of the mitochondrial outer membrane during apoptosis leads to the decay of mRNAs and polyadenylated noncoding RNAs. hfq, host factor for phage Q_β_; hPNPase, human PNPase; IMS, inner membrane space; MRP, mitochondrial RNA processing; mtRNA, mitochondrial RNA; PNPase, polynucleotide phosphorylase; Rsr, Ro sixty-related protein; sRNA, small regulatory RNA; SUV3, suppressor of Var 1, 3.

While PNPase can function independently as a 3ʹ to 5ʹ exoribonuclease, in bacteria, PNPase also serves as a component of an organized RNA degradation machine (Fig 2A). Termed the degradosome, this multiprotein complex is responsible for bulk mRNA decay [50, 51]. At the core of this RNA-degrading machine in gram-negative bacteria is the endoribonuclease RNase E, which initiates RNA decay. The essential N-terminal domain of RNase E contains the active site and additional features including the S1 domain and 5ʹ sensor pocket important for binding many RNAs (reviewed in [52]). The C-terminal domain is required for formation of the RNA degradosome and contains binding sites for other proteins; in the canonical *E*. *coli* RNA degradosome, these proteins include the glycolytic enzyme enolase, the DEAD-box RNA helicase RhlB and PNPase [53–55]. However, the RNase E–based degradosome can vary in composition between species or even within the same organism depending on cellular conditions [56]. In *C*. *crescentus*, aconitase is exchanged for enolase, and RNase D was validated as a legitimate degradosome component [57, 58]. Gram-positive bacteria have a similar RNA degradation machine that is likewise organized around a core endoribonuclease, in this case, RNase Y. Like RNase E, RNase Y has an unstructured C-terminal region with specific binding sites for PNPase, enolase, and an RNA helicase [59]. However, despite these functional similarities, the two proteins are evolutionarily distinct, belonging to different protein superfamilies [60]. Unlike RNase E, RNase Y features an N-terminal region with an RNA-binding KH domain and an active site–containing HD domain, and its C-terminal domain additionally interacts with 5ʹ to 3ʹ exoribonucleases [60, 61].

PNPase and the RNA degradosome of the gram-negative bacterium *E*. *coli* have been studied in much detail. As part of the degradosome, PNPase cooperates with RNase E to degrade a specific set of mRNAs, many of which encode proteins involved in macromolecule biosynthesis or modification [50]. Additionally, within the degradosome, binding of RhlB and PNPase to the RNase E scaffold is necessary for the degradation of highly structured RNA sequences, termed repeated extragenic palindrome (REP) elements that are found in some mRNAs [62–64].

Although PNPase participates in mRNA decay as a component of the RNA degradosome, the enzyme primarily functions independent of this machine. This is evident by the number and distribution of PNPase and RNase E molecules in *E*. *coli*. PNPase is approximately three to five times more abundant than RNase E and is mostly distributed throughout the cytoplasm (69% of PNPase; Fig 2A), whereas the majority of RNase E (91%) is located near or on the cell membrane [65]. Moreover, only a minority of *E*. *coli* PNPase trimers are bound to RNase E at any one time [53]. The independent function of PNPase is also evident by global gene expression profiling, which demonstrated that many mRNAs stabilized by the absence of PNPase are not significantly impacted by loss of the degradosome [50]. Furthermore, PNPase functions independently of the RNA degradosome in tRNA processing [37, 38, 66], rRNA degradation [40, 67], and sRNA-mediated gene regulation [68].

### A specialized function for PNPase in rRNA processing and decay

In bacteria, the fully assembled 70S ribosome is made up of the small 30S subunit containing the 16S rRNA and the large 50S subunit consisting of the 23S rRNA and 5S rRNA. In *E*. *coli* and presumably most gram-negative bacteria, PNPase and RNase R perform routine rRNA quality control by degrading fragments of 16S and 23S rRNAs that might otherwise compete with mature rRNAs for ribosomal proteins and impair proper ribosomal assembly [69, 70]. These rRNA fragments are generated by RNase E [70], which may cleave rRNAs that cannot be assembled into functional ribosomes due to improper processing, damage, or overabundance relative to ribosomal proteins. In *E*. *coli*, PNPase was not required for rRNA decay induced by nutrient starvation [71].

In some bacteria such as the radiation-resistant *D*. *radiodurans*, PNPase mediates rRNA degradation in response to nutrient starvation with the assistance of the Ro sixty-related protein, Rsr (Fig 2A) [72, 73]. Ro was originally identified in human cells as an autoantigen recognized by antibodies from lupus erythematosus patients [74]. Ro and its homologs, which are present in many vertebrates and in roughly 5% of bacterial genome sequences [75], bind to structured noncoding RNAs called Y-RNAs [13, 72, 74, 76, 77] and are involved in rRNA decay [73, 78, 79]. In *D*. *radiodurans*, Y-RNA tethers Rsr to PNPase resulting in the formation of the “RYPER” complex (Fig 2A) that degrades structured RNA including the 5S, 16S, and 23S rRNAs [72, 73]. Y-RNAs have a highly conserved structure that consists of an extended stem generated by pairing between bases at the 3ʹ and 5ʹ ends and a large internal loop that in many cases is decorated with two hairpins [80, 81]. A conserved region within the Y-RNA stem binds to Rsr, whereas a region containing two hairpins, one resembling a T-arm of a tRNA, binds to the KH and S1 domains of PNPase [72, 82]. The 3ʹ end of the misfolded rRNA appears to thread through the central pore of the toroid-shaped Rsr protein and into the central channel of PNPase, where it is degraded [72].

### The opposing functions of PNPase in regulating small RNAs

Although for many years it was largely assumed that PNPase only contributed to RNA processing and degradation in bacteria, it has become increasingly clear over the last decade that PNPase also plays an important role in regulating sRNA function and stability [68]. In bacteria, sRNAs range in size from 50 to 250 nucleotides and alter gene expression by sequestering regulatory proteins or by base-pairing with target mRNAs to modulate translation and transcript stability (reviewed in [83, 84]). Many sRNAs interact with RNA chaperones, such as FinO [85], ProQ [86, 87], or the host factor for phage Q_β_ (Hfq) [88, 89], to facilitate this process. In the latter case, Hfq protects sRNAs from degradation by occluding an endoribonuclease cleavage site [90, 91] and facilitates sRNA–mRNA annealing [92, 93].

In *E*. *coli* and its close relative *Salmonella* Typhimurium, binding by Hfq dictates whether an sRNA is degraded or stabilized by PNPase. For Hfq-independent sRNAs such as CopA, CsrB, and CsrC, PNPase degrades these RNAs following initial cleavage by RNase E [94, 95]. Even some Hfq-binding sRNAs such as SraL, RybB, and MicA are destabilized by PNPase [94, 96]; however, PNPase degrades only the pool of MicA that is not bound by Hfq [97, 98]. Indeed, PNPase binds and rapidly degrades several Hfq-binding sRNAs in vitro but only in the absence of Hfq [34]. In the presence of Hfq, PNPase instead forms a stable ternary complex with Hfq and sRNAs, and experimental evidence supports the existence of this complex in *E*. *coli* [34]. In vivo, PNPase stabilizes many Hfq-dependent sRNAs, and deletion of the gene encoding this RNase paradoxically results in reduced sRNA stability [34, 68, 97, 98].

What is the role of PNPase in facilitating sRNA-mediated gene regulation? In our speculative model, PNPase forms a complex with Hfq that is mediated by sRNAs (Fig 2A). Within this complex, PNPase is unable to degrade the sRNA because its 3ʹ end is bound to Hfq. After sRNA–mRNA pairing, Hfq is released from the complex and in most cases each RNA is first cleaved by an endoribonuclease (RNase E), followed by rapid degradation of the resulting sRNA and mRNA fragments by PNPase. In the absence of PNPase, specific mRNA fragments accumulate and go on to pair with additional sRNAs resulting in their cleavage by RNase E. By this mechanism, these mRNA fragments act to deplete the pool of specific sRNAs, resulting in decreased regulation of their mRNA targets.

Given that Hfq-binding sRNAs in *E*. *coli* and other gram-negative bacteria regulate many physiological processes—including DNA repair [99, 100], motility [101–104], biofilm formation [102, 105–111], and antibiotic resistance [112–114]—and that PNPase regulates sRNA stability and function, we postulate that the majority of the phenotypes associated with the loss of functional PNPase are due to its role in degrading or stabilizing sRNAs. There is already some evidence supporting this hypothesis. For example, the reduced rate of spontaneous mutation observed for an *E*. *coli pnp* deletion strain [115] may originate from reduced ArcZ sRNA levels, as disruption of the negative regulation of *mutS* by ArcZ also reduces the spontaneous mutation rate in *E*. *coli* [100]. Similarly, the role of PNPase in promoting biofilm formation may be due to its function in stabilizing Hfq-dependent sRNAs; recent studies have collectively shown that deletion of *pnp*, *hfq*, or genes encoding the sRNAs DsrA or ArcZ from *E*. *coli* each resulted in defects in biofilm formation [108, 116].

### Humans have a PNPase too

Studies of the mammalian PNPase have been fraught with controversy, and many functions have been reported for the human PNPase (hPNPase), including mitochondrial RNA import, processing, and decay and miRNA and mRNA degradation (Fig 2B). Careful studies mapping the cellular location of hPNPase indicate that it mainly resides in the mitochondrial inner membrane space (IMS) located between the outer and inner membranes [117, 118]. hPNPase is guided to the IMS via a mitochondrial targeting sequence that is cleaved off when it is translocated into the IMS [118]. Upon overexpression, hPNPase accumulates in other cellular compartments such as the cytoplasm [119], but conditions in which the natively expressed hPNPase is found in this space have not been identified until recently. A newly published study revealed that natively expressed hPNPase can also be released into the cytoplasm upon mitochondrial outer membrane permeabilization during programmed cell death, whereupon hPNPase contributes to global apoptotic RNA decay by degrading mRNAs and polyadenylated noncoding RNAs (Fig 2B) [120].

Within the IMS, PNPase is a peripheral membrane protein that reportedly binds the 5S rRNA and the *RNase P* and *RNase MRP* RNAs to facilitate importation of these RNAs into the mitochondrial central space, or matrix [25]. This import function of PNPase did not appear to require its catalytic activity, and intriguingly, a 20-nucleotide stem loop structure found in the *RNase P* and *MRP* RNAs was sufficient for PNPase-mediated mitochondrial import [25]. In addition, Wang and colleagues [25] found that processing of polycistronic tRNA transcripts in vivo required the *RNase P* RNA. These results appear to conflict with previous work showing that the *MRP* RNA was undetectable [121] or at infinitesimal levels [122] in HeLa cell mitochondria, that only a very small number of *RNase P* RNA molecules were associated with the mitochondria of HeLa cells [122, 123], and that a reconstituted mitochondrial RNase P lacking an RNA component was functional in processing mitochondrial precursor tRNAs [124]. As argued by Wang and colleagues [25], it is possible that RNase P exists in mammalian mitochondria in both the protein-only and H1 RNA-containing forms and that the RNA-containing form of RNase P is much less abundant.

Both RNases serve critical roles in mitochondria, in which RNase MRP cleaves the RNA primers used for mitochondrial DNA replication and RNase P processes the large mitochondrial polycistronic transcripts that give rise to 22 tRNAs, 12S and 16S rRNAs, as well as 13 mRNAs encoding electron transport chain (ETC) components involved in oxidative phosphorylation (reviewed in [125]), i.e., the synthesis of ATP that is powered by the transfer of electrons from NADH or FADH_2_ to O_2_. Likewise, a stable PNPase knockout in mouse embryonic fibroblasts resulted in the loss of both mitochondrial DNA and cellular respiration, supporting a role for PNPase in mitochondrial DNA maintenance [126]. The vital function of hPNPase in facilitating proper expression of the ETC components is further evidenced by the fact that knockdown of PNPase in HEK293 cells leads to impairment of the ETC and disruption of oxidative phosphorylation [117]. Additionally, several recent clinical reports demonstrate that patients suffering from hereditary hearing loss, delayed myelination, axonal neuropathy, and Leigh syndrome have mutations in *PNPT1*, the gene encoding hPNPase [26–29, 127]. In several of these reports, the authors provided evidence that the hPNPase encoded in these patients’ genomes contributes to a defect in oxidative phosphorylation and mitochondrial RNA import [27–29].

hPNPase also catalyzes mitochondrial RNA decay with assistance from the suppressor of Var 1, 3 (SUV3) RNA helicase [128–130]. The involvement of hPNPase in this process requires that it associate with the SUV3 helicase in the mitochondrial matrix. Consistent with some hPNPase binding SUV3 in the mitochondrial matrix, PNPase coimmunoprecipitated with SUV3 from mitochondrial cell extracts and foci of exogenously produced hPNPase and SUV3 colocalized with mitochondrial DNA and RNA [128, 130]. Furthermore, knockdown of hPNPase in HeLa or T-Rex 293 cells resulted in stabilization of mitochondrial mRNAs [128, 129], and depletion of hPNPase or SUV3 led to accumulation of mitochondrial double-stranded RNA [131].

hPNPase also facilitates degradation of the c-myc mRNA [132, 133] and miRNAs including miR-221, miR-222, and miR-106b in vitro and in vivo upon overexpression [134]. miRNAs are a class of sRNAs in humans that regulate gene expression by base-pairing with target mRNAs (reviewed in [135]). However, to degrade these RNAs, hPNPase must reside in the cytoplasm, but this has only been shown to occur during apoptosis or upon exogenous overexpression in human cells [119, 120, 136]. Thus, the role of PNPase in degrading these RNAs may not be relevant under most physiological conditions. Aside from this potential degradation role, PNPase also appears to facilitate the import of miR-378 into mitochondria, resulting in down-regulation of the *mt-ATP6* transcript and a reduction in ATP synthase activity [137].

## Conclusions

Although PNPase has been studied for over 60 years, new functions for this old enzyme have been recently uncovered. In bacteria, a novel function for PNPase in degrading some sRNAs and protecting others has been discovered [68, 94, 138]. Because each sRNA can potentially regulate hundreds of distinct transcripts, PNPase impacts many, if not most, regulatory circuits in bacterial cells. Therefore, we postulate that the vast majority of phenotypes associated with loss of PNPase function in bacteria are due to its role in mediating sRNA stability. Equally exciting were the discoveries that hPNPase mediates the importation of RNA into the mitochondrial matrix [25] and degrades mRNAs and polyadenylated noncoding RNAs upon release into the cytoplasm following mitochondrial outer membrane permeabilization during apoptosis [120]. Given the recent discovery that PNPase is critical for mitochondrial DNA maintenance [126], hPNPase is vital to the proper replication and function of mitochondria and to human life. Considering that these recent discoveries of additional functions for PNPase were made after more than a half century of study, we expect to see many more exciting findings reported on this ancient enzyme in the years to come.
